# Anti-centromere antibody exhibits specific distribution levels among anti-nuclear antibodies and may characterize a distinct subset in rheumatoid arthritis

**DOI:** 10.1038/s41598-017-07137-4

**Published:** 2017-07-31

**Authors:** Nobuo Kuramoto, Koichiro Ohmura, Katsunori Ikari, Koichiro Yano, Moritoshi Furu, Noriyuki Yamakawa, Motomu Hashimoto, Hiromu Ito, Takao Fujii, Kosaku Murakami, Ran Nakashima, Yoshitaka Imura, Naoichiro Yukawa, Hajime Yoshifuji, Atsuo Taniguchi, Shigeki Momohara, Hisashi Yamanaka, Fumihiko Matsuda, Tsuneyo Mimori, Chikashi Terao

**Affiliations:** 1 Department of Rheumatology and Clinical Immunology, Kyoto University Graduate School of Medicine, Kyoto, Japan; 2 Tokyo Woman’s Medical University, Tokyo, Japan; 3 Department of the Control for Rheumatic Disease, Kyoto University Graduate School of Medicine, Kyoto, Japan; 4 Department of Center for Genomic Medicine, Kyoto University Graduate School of Medicine, Kyoto, Japan; 5 Center for the Promotion of Interdisciplinary Education and Research, Kyoto University Graduate School of Medicine, Kyoto, Japan; 6 Division of Rheumatology, Immunology, and Allergy, Brigham and Women’s Hospital, Harvard Medical School, Boston, MA 02115 USA; 7 Division of Genetics, Brigham and Women’s Hospital, Harvard Medical School, Boston, MA 02115 USA; 8 Program in Medical and Population Genetics, Broad Institute, Cambridge, MA 02142 USA

## Abstract

Anti-centromere antibody (ACA) is one of the classical anti-nuclear antibody (ANA) staining patterns. However, characteristics of ACA in comparison with the other ANA patterns and clinical features of ACA-positive subjects have not been elucidated. Here, we examined all ANA patterns by indirect immunofluorescence for 859 rheumatoid arthritis (RA) patients. Together with the ANA data of 9,575 healthy volunteers, we compared distributions of the ANA levels. ACA was the only ANA that demonstrated a definite bimodal distribution of levels. ACA showed significantly higher levels than the other ANA staining patterns in both RA and healthy population (p < 0.0001). ACA-positivity was associated with old age and was observed more in females. We further recruited another cohort of 3,353 RA patients and confirmed the findings. ACA was also associated with Raynaud’s phenomenon (p = 6.8 × 10^−11^) in RA. As a conclusion, ACA displays a specific ANA staining pattern with a bimodal distribution, and ACA-positive RA may constitute a distinct subset with specific clinical features.

## Introduction

Anti-centromere antibody (ACA) is a type of anti-nuclear antibody (ANA) detected by indirect immunofluorescence displaying a centromeric pattern (discrete speckled pattern)^1^. Although ACA has been considered specific for limited cutaneous systemic sclerosis (SSc)^2^, it is occasionally detected in patients with primary biliary cirrhosis (PBC) and primary Sjögren’s syndrome (SS) with various range of positivity (11 to 27% and 3.7 to 27%, respectively)^3–5^. In some reports, a subset of patients with rheumatoid arthritis (RA) were also positive for ACA^6, 7^, as well as healthy subjects^8^.

We previously quantified ANA in 9,575 healthy subjects without connective tissue diseases and reported that 87 individuals (0.91%) of healthy subjects were positive for ACA^8^. ACA was associated with old age and was observed more in females in the healthy subjects. After reporting these findings, we noticed that for other ANA staining patterns most of Ab-positive subjects were enriched by less than 1:160, but subjects positive for ACA were enriched at higher titer levels, by more than 1:640. Thus, we hypothesized that ACA might exhibit a specific distribution where most ACA-positive subjects had high titer levels. Some previous studies have reported that patients with rheumatic diseases might have high levels of ACA^9^, but they had no comprehensive data for ANA and failed to reach a clear conclusion supported by statistical significance.

In this study, we compared the distribution level of ACA with that of the other staining patterns, namely, speckled, homogeneous, nucleolar and cytoplasmic patterns in healthy subjects, and in RA patients. We also analyzed correlates and clinical features of ACA-positivity in patients with RA.

## Results

### ACA demonstrates higher levels than the other ANA staining patterns and shows a bimodal distribution

**Figure 1:**
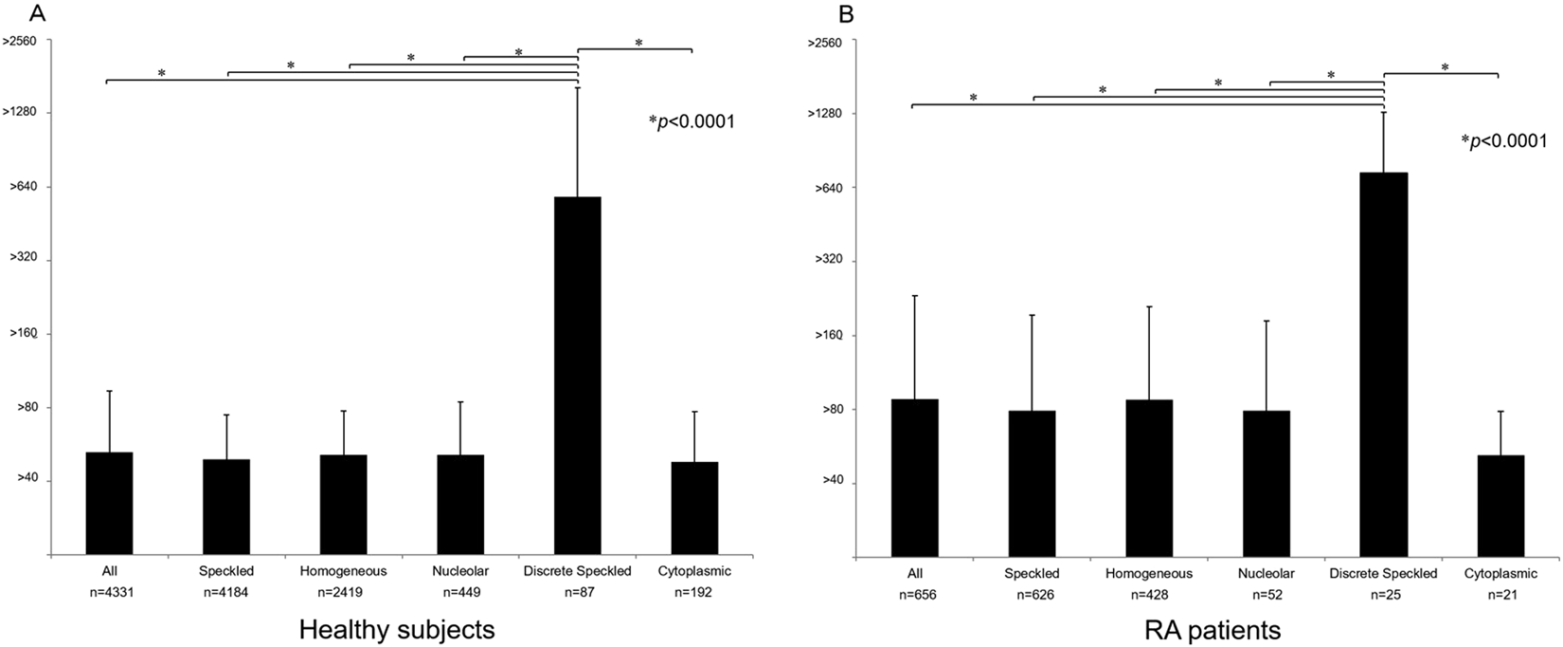
Distribution of ANA levels by each staining pattern. The ANA levels were log-transformed (log_2_(level/40) + 1) and compared by Wilcoxon rank sum test. ACA showed a significantly higher level than the other staining patterns in (**A**) healthy subjects, and (**B**) RA patients in the KURAMA cohort (p < 0.0001). Most subjects were positive for multiple ANA staining patterns.

We took advantage of the ANA data of 9,575 subjects from our previous report and analyzed the distribution of ANA levels in each staining pattern. In the 4,332 healthy subjects who were positive for ANA (Fig. 1A), only the discrete speckled pattern (ACA) showed a high-level-skewed distribution (Supplementary Figure 1A). When we compared the levels between all the staining patterns, ACA showed higher levels than all other staining patterns (p < 0.0001, Fig. 1A).

**Figure 2:**
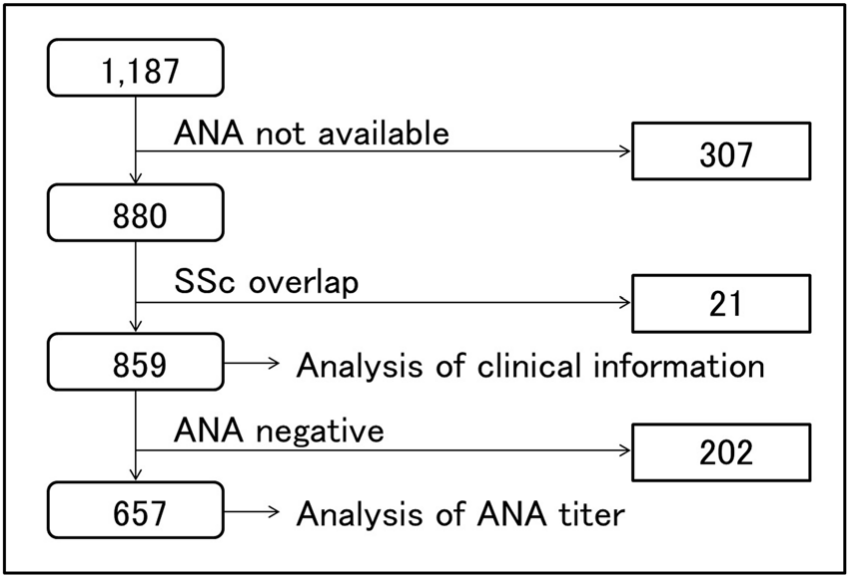
A flow chart of study participants with RA in the KURAMA cohort. From 1,187 RA patients, 307 patients without ANA data were excluded. Then we excluded 21 patients overlapping with SSc. The remaining 859 patients were used to compare ACA-positive patients with ACA-negative patients. We analyzed 657 subjects positive for ANA to compare the level of ANA among each staining pattern.

We confirmed this finding in patients with RA using data of 859 RA patients in the Kyoto University Rheumatoid Arthritis Management Alliance (KURAMA) cohort without SSc (p < 0.0001, Fig. 1B and Fig. 2). 657 subjects (76.5%) were positive for ANA and 25 patients (2.9%) were ACA-positive. 96.0% of RA patients positive ACA showed levels of 1:320 or higher, and less than 14.1% of patients with RA positive for the other staining patterns showed the comparable levels (Supplementary Figure 1B). Chi-squared test between ACA and speckled pattern which is the most representative staining pattern of ANA also showed significant high-level-skewed distribution of ACA in both healthy subjects and RA patients (Supplementary Table 1). Sixteen out of the 25 patients had data of enzyme-linked immunosorbent assay (ELISA) for ACA and all were positive. This distribution was consistent regardless of sex in the healthy population (Supplementary Figure 2). Only one male was positive for ACA in the RA subjects.

### Clinical features of ACA-positive RA patients

We analyzed the clinical features of ACA-positive patients with RA (Fig. 2 and Table 1). The ACA-positive group was older and tended to be prevalently female (96.0% vs 78.7%) (p = 0.0042 and 0.042, respectively, Table 1). We confirmed the association between ACA and old age in female RA subjects (Supplementary Table 2). ACA positivity was associated with low positivity of rheumatoid factor (RF) and anti-cyclic citrullinated protein antibody (ACPA), representative antibodies found in patients with RA (Table 1). No significant differences were observed in other clinical manifestations.

**Table 1:** Clinical features of ACA-positive RA patients.

	KURAMA				IORRA				KURAMA + IORRA			
	ACA + (n = 25)	ACA − (n = 834)	P Value	OR (95% CI)	ACA + (n = 61)	ACA − (n = 3292)	P Value	OR (95% CI)	ACA + (n = 86)	ACA − (n = 4126)	P Value	OR (95% CI)
Age	68.8 ± 11.9	61.2 ± 14.2	0.0042	0.15 (0.0086–0.73)	64.8 ± 13.8	57.1 ± 14.7	6.4 × 10^−5^		65.9 ± 13.3	57.9 ± 14.7	6.4 × 10^−7^	0.19 (0.048–0.52)
Male, %	4.00	21.3	0.042		3.28	14.2	0.0089	0.20 (0.033–0.66)	3.49	15.7	0.00070	
Age at onset	56.6 ± 16.2	49.4 ± 16.0	0.038		46.0 ± 15.6	45.3 ± 15.0	0.75		49.2 ± 17.1	46.2 ± 15.3	0.085	
RA duration	11.9 ± 8.2	11.3 ± 10.7	0.74		18.6 ± 13.6	11.6 ± 10.8	2.8 × 10^−5^		16.6 ± 12.6	11.6 ± 10.8	5.5 × 10^−5^	
RF, %	56.0	76.7	0.0029	0.39 (0.17–0.89)	83.6	84.0	0.86	0.94 (0.51–2.05)	75.6	82.5	0.097	0.66 (0.41–1.11)
ACPA, %	66.7	78.1	0.21	0.56 (0.24–1.41)	86.8	80.9	0.38	1.56 (0.75–3.79)	80.5	80.3	0.95	1.02 (0.59–1.86)
Stage	2.89 ± 1.36	2.52 ± 1.17	0.38		NA	NA			NA	NA		
Class	2.11 ± 0.78	1.85 ± 0.77	0.35		NA	NA			NA	NA		
TSS	174.5 ± 149.1	103.3 ± 102.1	0.35		11.5 ± 15.2	22.6 ± 24.8	0.58		35.4 ± 79.5	44.5 ± 67.4	0.24	
DAS28 ESR	4.34 ± 1.93	2.97 ± 1.27	0.086		NA	NA			NA	NA		
MTX, %	60.0	66.5	0.52	0.76 (0.34–1.76)	55.7	69.6	0.025	0.55 (0.33–0.92)	57.0	69.0	0.018	0.60 (0.39–0.92)
Bio, %	64.0	40.2	0.022	2.65 (1.18–6.32)	31.1	28.1	0.66	1.15 (0.65–1.97)	40.7	30.6	0..044	1.56 (1.00–2.40)

KURAMA, Kyoto University Rheumatoid Arthritis Management Alliance; IORRA, Institute of Rheumatology, Rheumatoid arthritis; ACA, anti-centromere antibody; OR, odds ratio; 95% CI, 95% confidence interval; RA, rheumatoid arthritis; TSS, total sharp score; DAS, disease activity score; ESR, erythrocyte sedimentation rate; MTX, methotrexate; Bio: biologic therapy; NA, not applicable. mean ± standard deviation is indicated for quantitative traits.

We analyzed 3,353 RA patients without SSc whose ANA data were available from the Institute of Rheumatology, Rheumatoid Arthritis (IORRA) cohort to confirm the associations. 61 subjects of the 3,353 patients (1.8%) were positive for ACA. The association between ACA-positivity and old age and females were replicated (p = 6.4 × 10^−5^ and p = 0.0089, respectively, Table 1). The associations between ACA and RA-related autoantibodies were not replicated (Table 1).

We confirmed these observations in the analysis of ANA-positive RA (Table 2).

**Table 2:** Clinical features of ACA-positive RA patients among ANA-positive subjects.

	KURAMA				IORRA				KURAMA + IORRA			
	ACA + (n = 25)	ACA – ANA + (n = 632)	P Value	OR (95% CI)	ACA + (n = 61)	ACA − ANA + (n = 2313)	P Value	OR (95% CI)	ACA + (n = 86)	ACA − ANA + (n = 2945)	P Value	OR (95% CI)
Age	68.8 ± 11.9	60.7 ± 14.2	0.0026		64.8 ± 13.8	56.1 ± 14.8	8.9 × 10^−6^		65.9 ± 13.3	57.1 ± 14.7	6.1 × 10^−8^	
Male, %	4.00	19.1	0.065	0.17 (0.010–0.82)	3.28	10.8	0.058	0.28 (0.046–0.91)	3.49	12.6	0.012	0.25 (0.062–0.68)
Age at onset	56.6 ± 16.2	49.6 ± 15.6	0.033		46.0 ± 16.6	44.1 ± 14.9	0.36		49.2 ± 17.1	45.1 ± 15.2	0.021	
RA duration	11.9 ± 8.2	11.6 ± 10.4	0.64		18.6 ± 13.5	11.8 ± 10.9	5.0 × 10^−5^		16.6 ± 12.6	11.8 ± 10.8	0.00014	
RF, %	56.0	81.3	0.0011	0.30 (0.13–0.69)	83.6	88.5	0.24	0.66 (0.34–1.40)	75.6	87.0	0.0023	0.46 (0.29–0.79)
ACPA, %	66.7	82.0	0.088	0.48 (0.20–1.20)	86.8	86.0	0.86	0.93 (0.38–1.96)	80.5	85.0	0.27	0.73 (0.42–1.33)
TSS	174.5 ± 149.1	105.5 ± 104.3	0.36		11.5 ± 15.2	23.6 ± 24.6	0.70		35.5 ± 79.5	48.2 ± 71.4	0.37	
MTX, %	60.0	67.9	0.47	0.74 (0.33–1.73)	55.7	69.1	0.027	0.56 (0.34–0.95)	57.0	68.8	0.020	0.60 (0.39–0.93)
Bio, %	64.0	45.4	0.035	2.39 (1.06–5.73)	31.1	28.9	0.71	0.90 (0.53–1.59)	40.7	32.5	0.11	1.43 (0.91–2.20)

KURAMA, Kyoto University Rheumatoid Arthritis Management Alliance; IORRA, Institute of Rheumatology, Rheumatoid arthritis; ACA, anti-centromere antibody; OR, odds ratio; 95% CI, 95% confidence interval; RA, rheumatoid arthritis; TSS, total sharp score; DAS, disease activity score; ESR, erythrocyte sedimentation rate; MTX, methotrexate; Bio: biologic therapy. mean ± standard deviation is indicated for quantitative traits.

We further investigated the associations between ACA-positivity and complications observed in RA. Raynaud’s phenomenon and secondary SS were significantly associated with ACA-positivity (p = 6.8 × 10^−11^ and 0.0047, OR:19.7 (95% CI:8.55–46.7) and 7.37 (2.02–21.5), respectively, Table 3). Logistic regression analysis with response variate of ACA and covariates of age and sex confirmed significant associations between ACA and Raynaud’s phenomenon and secondary SS (Supplementary Table 3). Interstitial pneumonia and complication of PBC showed tendency of associations with ACA-positivity (Table 3). Again, we confirmed these observations in the analysis of ANA-positive RA (Table 4). These findings indicate that ACA-positive RA patients tend to present with phenotypes related with SSc, even if they do not suffer from SSc. We did not have sufficient data concerning other SSc-associated phenotypes. There was no clinical information available from the IORRA cohort.

**Table 3:** Clinical manifestations and complications in ACA-positive RA patients.

	ACA + (n = 25)	ACA − (n = 834)	p Value	OR (95% CI)
Raynaud’s phenomenon, %	56.0	6.06	6.8 × 10^−11^	19.7 (8.55–46.7)
Interstitial pneumonia, %	20.0	10.6	0.18	2.12 (0.69–5.39)
Secondary SS, %	16.0	2.52	0.0047	7.37 (2.02–21.5)
PBC, %	4.0	0.25	0.087	17.0 (0.77–182.9)

ACA, anti-centromere antibody; OR, odds ratio; 95% CI, 95% confidence interval; SS, Sjögren’s syndrome; PBC, Primary biliary cirrhosis.

**Table 4:** Clinical manifestations and complications in ACA-positive RA patients among ANA-positive RA subjects.

	ACA + (n = 25)	ACA − ANA + (n = 632)	p Value	OR (95% CI)
Raynaud’s phenomenon, %	56.0	7.8	6.86 × 10^−16^	15.3 (6.62–36.4)
Interstitial pneumonia, %	20.0	10.8	0.16	2.07 (0.67–5.31)
Secondary SS, %	16.0	3.3	0.0036	5.54 (1.52–16.2)
PBC, %	4.0	0.32	0.041	12.8 (0.58–137.6)

ACA, anti-centromere antibody;OR, odds ratio; 95% CI, 95% confidence interval; SS, Sjögren’s syndrome; PBC: Primary biliary cirrhosis.

### Analysis of HLA-DRB1 alleles susceptible to ACA positivity

HLA-DRB1 is the strongest susceptibility gene to RA^10^. We searched for HLA-DRB1 alleles associated with ACA-positivity in a total of 23 ACA-positive and 818 ACA-negative RA. The combined analysis of the two cohorts showed that DRB1*10:01 tended to be associated with ACA-positivity, although it did not reach a significant level after Bonferroni’s correction (p = 0.034, Table 5). Imputation of HLA-DRB1 alleles in our previous study using healthy subjects did not identify DRB1*10:01 as a significant allele (data not shown)^8^. Analysis on shared epitope (SE), a common allelic group showing associations with RA susceptibility, did not show a significant difference (Supplementary Table 4). When we analyzed HLA-DRB1 serotypes, we did not find significant associations (Supplementary Table 5). Power calculation suggested that HLA-DRB1 alleles with odds ratio of more than 5 and allele frequency of more than 0.1 were not likely (Supplementary Table 6).

**Table 5:** Association of HLA-DRB1 alleles with ACA-positivity.

	KURAMA				IORRA				KURAMA + IORRA			
	ACA + (n = 12)	ACA − (n = 348)	P Value	OR (95% CI)	ACA + (n = 11)	ACA − (n = 470)	P Value	OR (95% CI)	ACA + (n = 23)	ACA − (n = 818)	P Value	OR (95% CI)
DRB1*01:01	0.083	0.070	0.88	1.34 (0.20–5.29)	0.14	0.073	0.94	1.22 (0.18–4.74)	0.11	0.072	0.50	1.78 (0.58–4.56)
DRB1*04:01	0.00	0.033	0.48	0.00 (0.00–3.14)	0.045	0.028	0.39	0.00 (0.00–2.94)	0.022	0.030	0.94	0.78 (0.043–3.84)
DRB1*04:03	0.042	0.02	0.51	2.17 (0.11–12.4)	0.00	0.016	0.56	0.00 (0.00–6.54)	0.022	0.018	0.95	1.33 (0.073–5.71)
DRB1*04:04	0.00	0.0029	0.71	0.00 (0.00–49.1)	0.00	0.0043	0.75	0.00 (0.00–25.5)	0.00	0.0037	0.68	0.00 (0.00–13.8)
DRB1*04:05	0.29	0.26	0.84	1.03 (0.29–3.48)	0.41	0.27	0.19	1.11 (0.31–4.06)	0.35	0.27	0.17	1.16 (0.47–2.87)
DRB1*04:06	0.042	0.019	0.48	2.34 (0.12–13.5)	0.045	0.021	0.035	4.74 (0.70–19.6)	0.043	0.020	0.27	2.27 (0.35–8.18)
DRB1*04:07	0.042	0.0057	0.14	7.82 (0.38–58.6)	0.00	0.0021	0.82	0.00 (0.00–66.2)	0.022	0.0037	0.060	6.15 (0.32–38.1)
DRB1*04:10	0.042	0.019	0.48	2.34 (0.12–13.5)	0.045	0.019	0.43	2.28 (0.12–12.7)	0.043	0.019	0.23	2.42 (0.38–8.76)
DRB1*08:01	0.00	0.0014	0.79	0.00 (0.00–175.0)	0.00	0.00	—	—	0.00	0.00061	0.87	0.00 (0.00–211.0)
DRB1*08:02	0.00	0.023	0.29	0.00 (0.00-3.82)	0.00	0.014	0.56	0.00 (0.00–6.54)	0.00	0.018	0.36	0.00 (0.00–2.45)
DRB1*08:03	0.13	0.050	0.26	3.39 (0.72–12.0)	0.045	0.041	0.99	1.06 (0.057–5.68)	0.087	0.045	0.30	2.31 (0.66–6.37)
DRB1*09:01	0.17	0.16	0.59	1.37 (0.36–4.45)	0.091	0.19	0.039	0.00 (0.00–0.55)	0.13	0.18	0.57	0.94 (0.34–2.29)
DRB1*10:01	0.083	0.0072	0.024	22.9 (2.80-154.1)	0.00	0.0053	0.72	0.00 (0.00–19.4)	0.043	0.00061	0.034	9.63 (1.40–41.4)
DRB1*11:01	0.00	0.02	0.33	0.00 (0.00–4.43)	0.00	0.021	0.47	0.00 (0.00–4.08)	0.00	0.021	0.32	0.00 (0.00–2.07)
DRB1*12:01	0.042	0.029	0.72	1.49 (0.080–8.27)	0.00	0.028	0.39	0.00 (0.00–2.94)	0.043	0.028	0.53	1.60 (0.25–5.68)
DRB1*12:02	0.00	0.023	0.29	0.00 (0.00–3.82)	0.00	0.022	0.45	0.00 (0.00–3.87)	0.00	0.023	0.30	0.00 (0.00–1.89)
DRB1*13:01	0.00	0.0014	0.79	0.00 (0.00–175.0)	0.00	0.0011	0.87	0.00 (0.00–235.8)	0.00	0.0012	0.81	0.00 (0.00–58.8)
DRB1*13:02	0.00	0.034	0.46	0.00 (0.00–2.97)	0.00	0.034	0.65	0.00 (0.00–2.71)	0.022	0.034	0.90	0.73 (0.040–3.57)
DRB1*14:01	0.00	0.014	0.41	0.00 (0.00–6.44)	0.00	0.0085	0.65	0.00 (0.00–11.2)	0.00	0.011	0.47	0.00 (0.00–4.08)
DRB1*14:02	0.00	0.00	—	—	0.00	0.0011	0.87	0.00 (0.00–235.8)	0.00	0.00061	0.87	0.00 (0.00–211.0)
DRB1*14:03	0.00	0.011	0.46	0.00 (0.00–8.29)	0.00	0.014	0.56	0.00 (0.00–6.54)	0.00	0.013	0.44	0.00 (0.00–3.46)
DRB1*14:05	0.00	0.013	0.43	0.00 (0.00–7.25)	0.00	0.012	0.59	0.00 (0.00–7.86)	0.00	0.012	0.45	0.00 (0.00–3.64)
DRB1*14:06	0.00	0.011	0.46	0.00 (0.00–8.29)	0.00	0.013	0.87	0.00 (0.00–8.72)	0.00	0.012	0.76	0.00 (0.00–4.08)
DRB1*14:54	0.00	0.011	0.46	0.00 (0.00–8.29)	0.00	0.0064	0.025	0.12 (0.017–2.37)	0.00	0.0085	0.53	0.00 (0.00–5.35)
DRB1*15:01	0.00	0.056	0.26	0.00 (0.00–1.49)	0.00	0.048	0.14	0.28 (0.080–1.30)	0.00	0.051	0.28	0.00 (0.00–0.81)
DRB1*15:02	0.00	0.093	0.10	0.00 (0.00–0.89)	0.045	0.093	0.68	0.42 (0.023–2.21)	0.022	0.093	0.24	0.22 (0.012–1.05)
DRB1*16:02	0.042	0.0086	0.22	5.18 (0.26–34.2)	0.00	0.012	0.57	0.00 (0.00–7.14)	0.043	0.010	0.035	4.49 (0.68–17.1)

KURAMA, Kyoto University Rheumatoid Arthritis Management Alliance; IORRA, Institute of Rheumatology, Rheumatoid arthritis; ACA, anti-centromere antibody; OR, odds ratio; 95% CI, 95% confidence interval.

### Analysis of Total Sharp Score (TSS) data in the ACA-positive RA patients

Lastly, we analyzed an association between ACA and bone destruction in RA by multivariate analysis. While TSS in the ACA-positive group had a tendency to be high in the KURAMA cohort, we could not replicate the tendency in the IORRA cohort (Table 6).

**Table 6:** An association study between TSS and ACA.

	KURAMA				IORRA				KURAMA + IORRA			
	Estimate	Standard Error	t Value	P Value	Estimate	Standard Error	t Value	P Value	Estimate	Standard Error	t Value	P Value
ACA	94.0	21.0	2.24	0.027	−13.3	4.02	5.59	0.018	−12.9	9.49	1.36	0.17

KURAMA, Kyoto University Rheumatoid Arthritis Management Alliance; IORRA, Institute of Rheumatology, Rheumatoid arthritis; ACA, anti-centromere antibody; RA, rheumatoid arthritis; TSS, total sharp score.

## Discussion

There are some studies reporting observation of high levels of ACA^7, 9^ but to the best of our knowledge, none of the studies support their observation with statistical significance. There are a few published studies on the clinical aspects of ACA-positive patients^11^. ACA is reported to have high negative predictive value for CREST syndrome^12^, but it is a rarity to focus on relationship between ACA and SSc-related phenotypes in non-SSc patients. This is the first comprehensive study to statistically demonstrate the higher levels of ACA compared to the other staining patterns of ANA. ACA-positive subjects are generally older and prevalently female. We also revealed the clinical characteristics of ACA-positive patients of RA. ACA-positive RA patients presented with Raynaud’s phenomenon more frequently than ACA-negative RA patients. These findings suggest that ACA seldom presents as false-positive and could thus serve as a potential biomarker with high specificity. This study is a retrospective study and the limited number of subjects positive for ACA makes it difficult to argue clinical usefulness of the current findings. Future experimental validation and elucidation of mechanisms of ACA production together with prospective clinical studies would be necessary.

Our results suggest that ACA-positive RA might be a distinct subset. ACA is also frequently positive in patients with SSc and it is regarded as a subset of the disease. The onset age of ACA-positive SS is high^4^ and the patients of SS often have Raynaud’s phenomenon^4, 13–15^. Since these features are the same as the findings in the current study and SSc was not included in this study, these characteristics might be the common features of ACA-positive subjects. Interstitial pneumonia is often complicated with SSc. While there have not been any published reports showing that interstitial pneumonia is frequently seen in ACA-positive patients with SSc, our study suggested that we should be aware of interstitial pneumonia in ACA-positive RA patients even without SSc. This study also suggests the possibility that the phenotypes found in patients with limited SSc might be associated with the presence of ACA and not necessarily with SSc itself.

We failed to confirm that ACA-positivity is another risk factor of bone destruction. We could not find susceptibility HLA-DRB1 alleles to ACA positivity. Since this study is still underpowered especially for the number of ACA-positive RA subjects, we cannot draw any conclusions regarding associations between ACA positivity and joint destruction or HLA-DRB1 alleles. HLA-DRB1*04:05 is a susceptibility allele to ANA^8^ and exhibits strong association with the development of RA and joint destruction^10^. Thus, there is a possibility that ACA is associated with DRB1 alleles and joint destruction. Increasing the number of subjects would clarify these aspects in the future.

While experimental validation for the association between ACA and SSc-related phenotypes is beyond the scope of this study, it would be interesting to inject ACA purified from patients positive for ACA into experimental animals such as monkeys and observe whether they develop SSc-related phenotypes including Raynaud’s phenomenon.

## Conclusions

Our study demonstrated that the level of ACA showed a bimodal distribution and is significantly higher than the other staining patterns of ANA in both healthy subjects and RA patients. The current results also suggested that the ACA-positive RA patients may constitute a definite subset. We could not elucidate whether ACA-positivity is a risk factor of bone destruction. Further large-scale studies would be essential to identify specific susceptibility gene(s) to ACA-positivity.

## Patients and Methods

### Study Population

The data collection and statistical analyses in this study were approved by the ethic committee in Kyoto University (Kyoto 606-8507, Japan) and Tokyo Women’s University (Tokyo 162-8666, Japan) and adhered to the ethical guidelines for clinical studies in Japan.

A total of 9,575 Japanese healthy volunteers in our previous report in which we had excluded subjects suspected of having connective tissue diseases were recruited for analyzing the distribution of the levels of each ANA staining pattern^8^.

We screened 1,187 RA patients from the KURAMA cohort of Kyoto University for the availability of ANA data. 880 patients had ANA data, but we excluded 21 subjects since they fulfilled American college of Rheumatology (ACR) criteria for SSc in 1980 and ACR/European League Against Rheumatism (EULAR) criteria for SSc in 2013. As a result, 859 RA patients were included in the analysis (Fig. 2). When we compared the ANA level of each staining pattern, we analyzed 657 RA patients who were positive for ANA (details are shown in Fig. 2).

For validation study, we recruited 3,404 patients with RA whose ANA data were available from the IORRA cohort of Tokyo Woman’s Medical University. We excluded 51 patients who had possibilities of SSc. As a result, 3,353 patients were analyzed for the validation study.

All of the patients fulfilled the ACR revised criteria for RA in 1987^16^ or the ACR and EULAR classification criteria for RA in 2010^17, 18^.

Written informed consent was obtained from all of the study participants except for a limited number of the KURAMA subjects. For these patients, the information regarding the construction of the KURAMA database was disclosed instead of obtaining written informed consent as previously described^19^.

### Data Collection

Across the study, we used data of ANA which were detected by indirect immunofluorescence with HEp-2 cell lines as substrate at an initial serum dilution of 1:40 using FITC-conjugated goat anti-human immunoglobulins. Detection was performed in SRL.Inc or LSI Medience Corporation, both of which are representatives of the Japanese largest clinical laboratory testing companies^8^. For the RA patients in the KURAMA cohort, we obtained data of ANA with a data collecting system^20^ from medical records. For the RA patients in the IORRA cohort, we obtained ANA data from the database in which the patients’ clinical information were stored every six months. Detailed information of ANA levels in each staining pattern was not available in the IORRA cohort. When multiple ANA data were available for a patient, single ANA data was randomly chosen.

We also collected information of age, sex, age at onset of RA, RF, ACPA, Steinbrocker’s RA stage, class, DAS 28, TSS and use of methotrexate and biologic agents from the database in each cohort. The information of Steinbrocker’s stage, class, DAS 28 in the RA patients were only available in the KURAMA data^18^. We analyzed the associations between ACA and clinical features especially to find an ACA-specific effect on RA outcome^21, 22^.

We surveyed complications of PBC, secondary SS^23, 24^ and the existence of Raynaud’s phenomenon and interstitial pneumonia which were manifestations of SSc^25^ in the enrolled patients with RA based on the clinical chart review. The secondary SS was defined by complication with other rheumatic diseases including RA. The information of complications and manifestations were available only in the patients from the KURAMA cohort.

### TSS Data

Using conventional X-rays, TSS was scored by an experienced reader (MF) for patients in the KURAMA cohort, and two other experienced readers (SY and KY) for patients in the IORRA cohort, according to the Sharp/van der Heijde method with a total score range of 0 to 448^26^. The intraobserver correlation coefficients for the TSS in the KURAMA and the IORRA cohort were 0.93 and 0.95, respectively and interobserver correlation coefficients for the TSS in the IORRA cohort was 0.85^27^.

### HLA-DRB1 Genotyping

In the KURAMA cohort, data of HLA-DRB-1 typed with the WALKFlow system^28^ were available for 360 patients among 859 patients with RA. In the IORRA cohort, HLA-DRB1 genotyping data by a sequencing-based typing method using the AlleleSEQR HLA-DRB1 typing kit (Abbott, Tokyo, Japan) were available for the 481 patients. DRB1*01:01, *04:01, *04:04, *04:05, *04:10, *10:01, *14:02 and *14:06 were classified as SE which is a five amino acid sequence motif in residues at amino acid position 70–74 of the HLA-DRβ chain associated with RA onset and severe joint destruction.

### Statistical Analysis

The ANA levels were log-transformed (log_2_(level/40) + 1) and their distributions were compared by Wilcoxon rank sum test between ACA group and each of the other groups based on staining patterns in the healthy volunteers or the RA patients who were positive for ANA. We compared clinical and genetic characteristics between ACA-positive and -negative RA patients. We performed Wilcoxon signed-rank test for quantitative data, and Pearson’s chi-square test or Fisher’s exact test for binary data. Linear regression analysis with response variate of TSS and an independent variable of ACA with covariates of RA duration, gender, age and RF was performed by standard least squares. Odds ratios were also calculated with 95% confidence intervals.

The analyses were separately performed for RA patients in each cohort. We also performed the analyses in the combined study. Since TSS is influenced by various factors whose distribution was different between the cohorts, we put an indicator variable of cohort on TSS analysis in the combined study. We performed power calculation of the current study for the HLA analyses with use of ‘Genetics Design’ package of R software^29^.

Statistical analyses were performed using JMP pro version 12 or R statistical software. Significant level was determined by Bonferroni’s correction. When clinical information were available in both the KURAMA and IORRA cohorts, we regarded an association as significant only when it showed a consistent association pattern with p-value less than 0.05 in each cohort and overall p-value in the combined study less than the significant level.

## Electronic supplementary material

supplementary
