# Supplementary material for: Anti-centromere antibody exhibits specific distribution levels among anti-nuclear antibodies and may characterize a distinct subset in rheumatoid arthritis
Source: Sci Rep. 2017 Jul 31;7:6911. doi: 10.1038/s41598-017-07137-4 (PMC5537247; doi:10.1038/s41598-017-07137-4)
Supplement: Supplementary file 1 — supplementary [file 41598_2017_7137_MOESM1_ESM.doc]

**Anti-centromere antibody exhibits a specific distribution of titers among anti-nuclear antibodies and characterizes a distinct subset in rheumatoid arthritis**

Nobuo Kuramoto^1^, Koichiro Ohmura^1^, Katsunori Ikari^2^, Koichiro Yano^2^, Moritoshi Furu^3^, Noriyuki Yamakawa^1^, Motomu Hashimoto^3^, Hiromu Ito^3^, Takao Fujii^1^, Kosaku Murakami^1^, Ran Nakashima^1^, Yoshitaka Imura^1^, Naoichiro Yukawa^1^, Hajime Yoshifuji,^1^ Atsuo Taniguchi^2^, Shigeki Momohara^2^, Hisashi Yamanaka^2^, Fumihiko Matsuda^4^, Tsuneyo Mimori^1,3^, and Chikashi Terao^4,5,6,7,8^

^1^Department of Rheumatology and Clinical Immunology, Kyoto University Graduate School of Medicine, Kyoto, Japan; ^2^Tokyo Woman’s Medical University, Tokyo, Japan; ^3^Department of the Control for Rheumatic Disease, Kyoto University Graduate School of Medicine, Kyoto, Japan; ^4^Department of Center for Genomic Medicine; ^5^Center for the Promotion of Interdisciplinary Education and Research, Kyoto University Graduate School of Medicine, Kyoto, Japan; ^6^Division of Rheumatology, Immunology, and Allergy; ^7^Division of Genetics, Brigham and Women’s Hospital, Harvard Medical School, Boston, MA 02115, USA; ^8^Program in Medical and Population Genetics, Broad Institute, Cambridge, MA 02142, USA.

**Supplementary Figure Legend**

**Supplementary Figure 1. Rate of ANA levels by each staining pattern.**

We displayed the rate of ANA levels by each staining pattern, coloring the titer levels. ACA has significant higher rate of high ANA level subjects in (A) healthy volunteers, and (B) RA patients in KURAMA cohort.

**Supplementary Figure 2. ANA levels by each staining pattern separated by sex.**

In healthy subjects, the distribution of ANA levels were similar in both (A) females, and (B) males.

**Supplementary Figure 1**


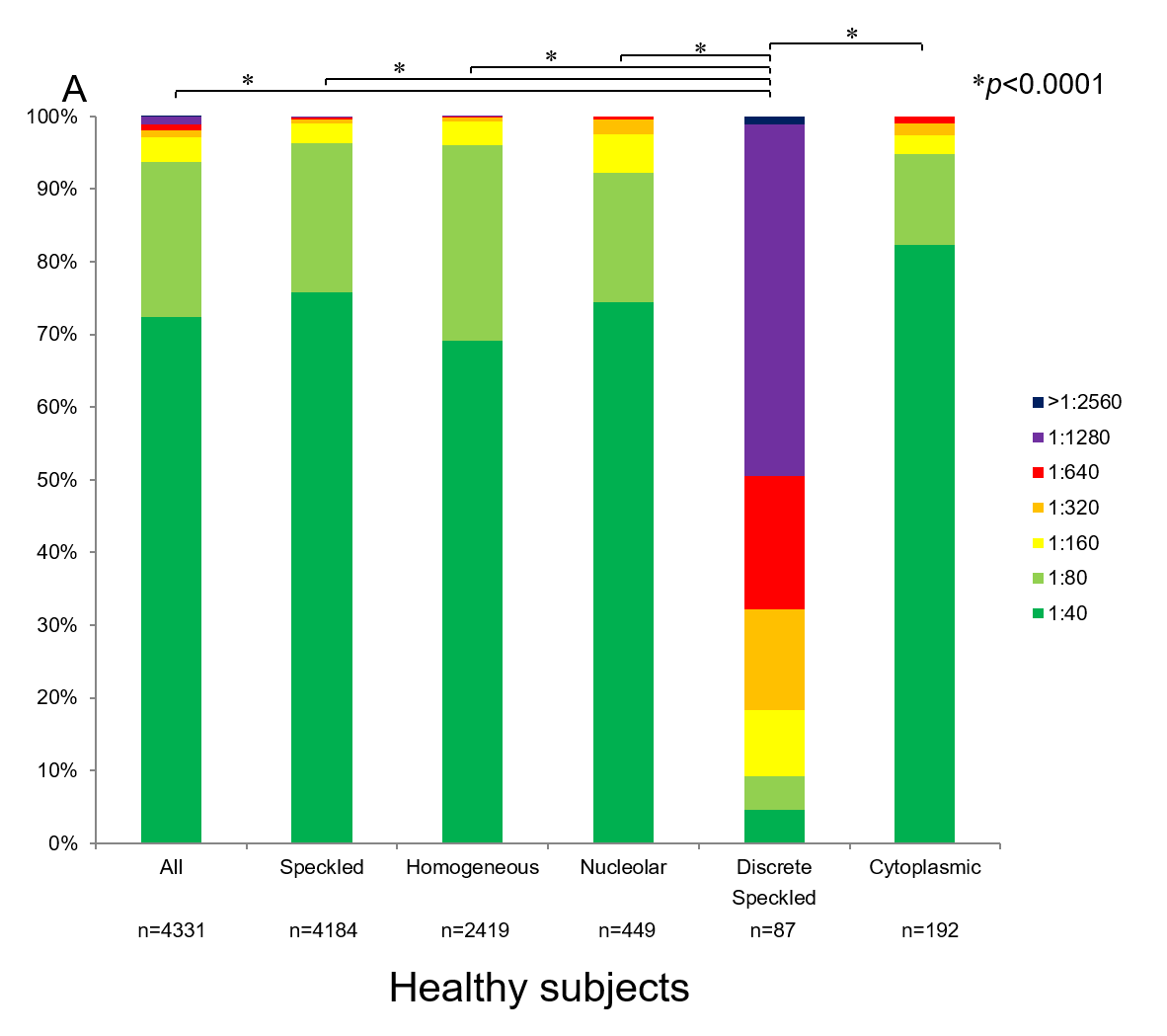


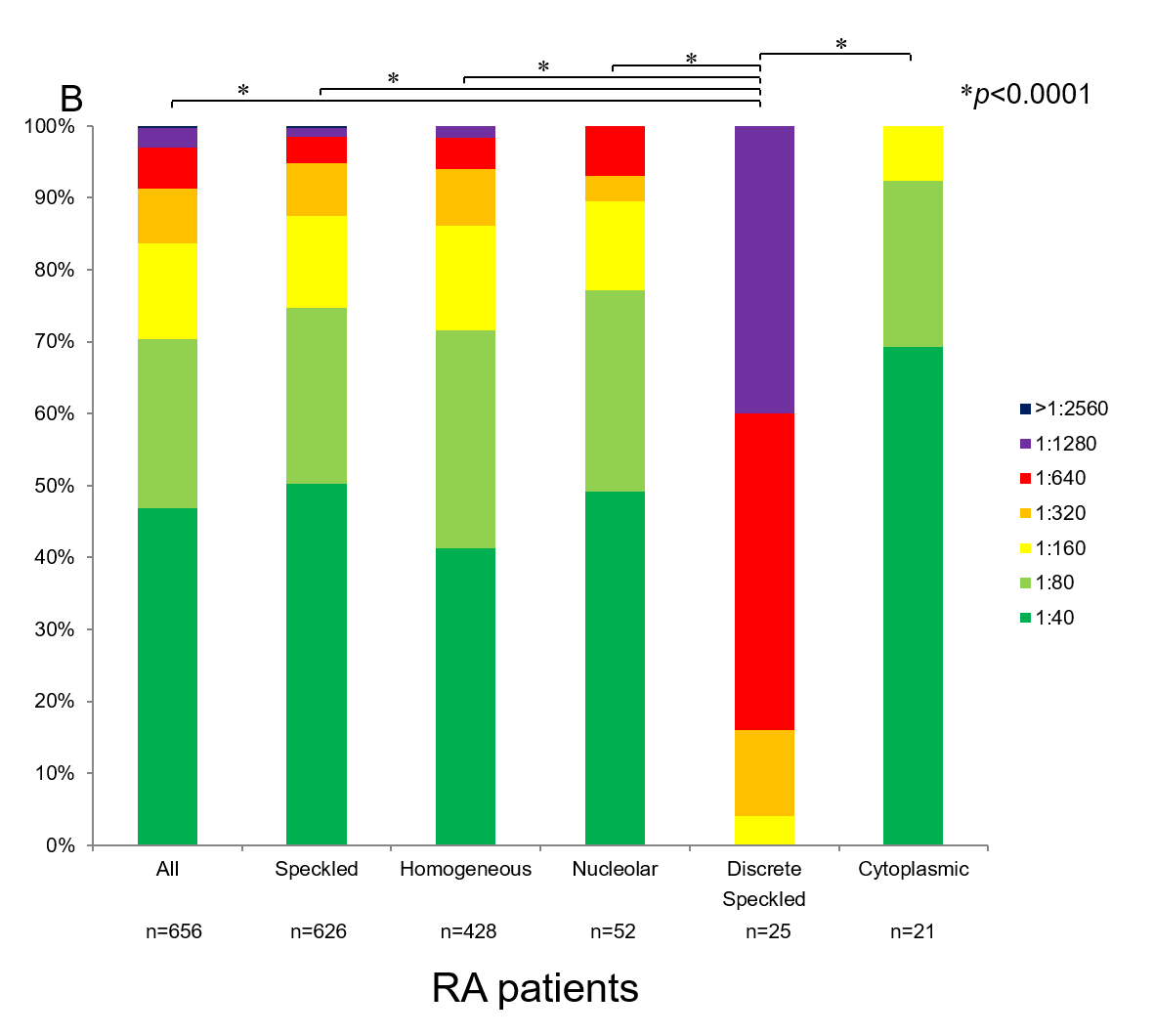


**Supplementary Figure 2**

**
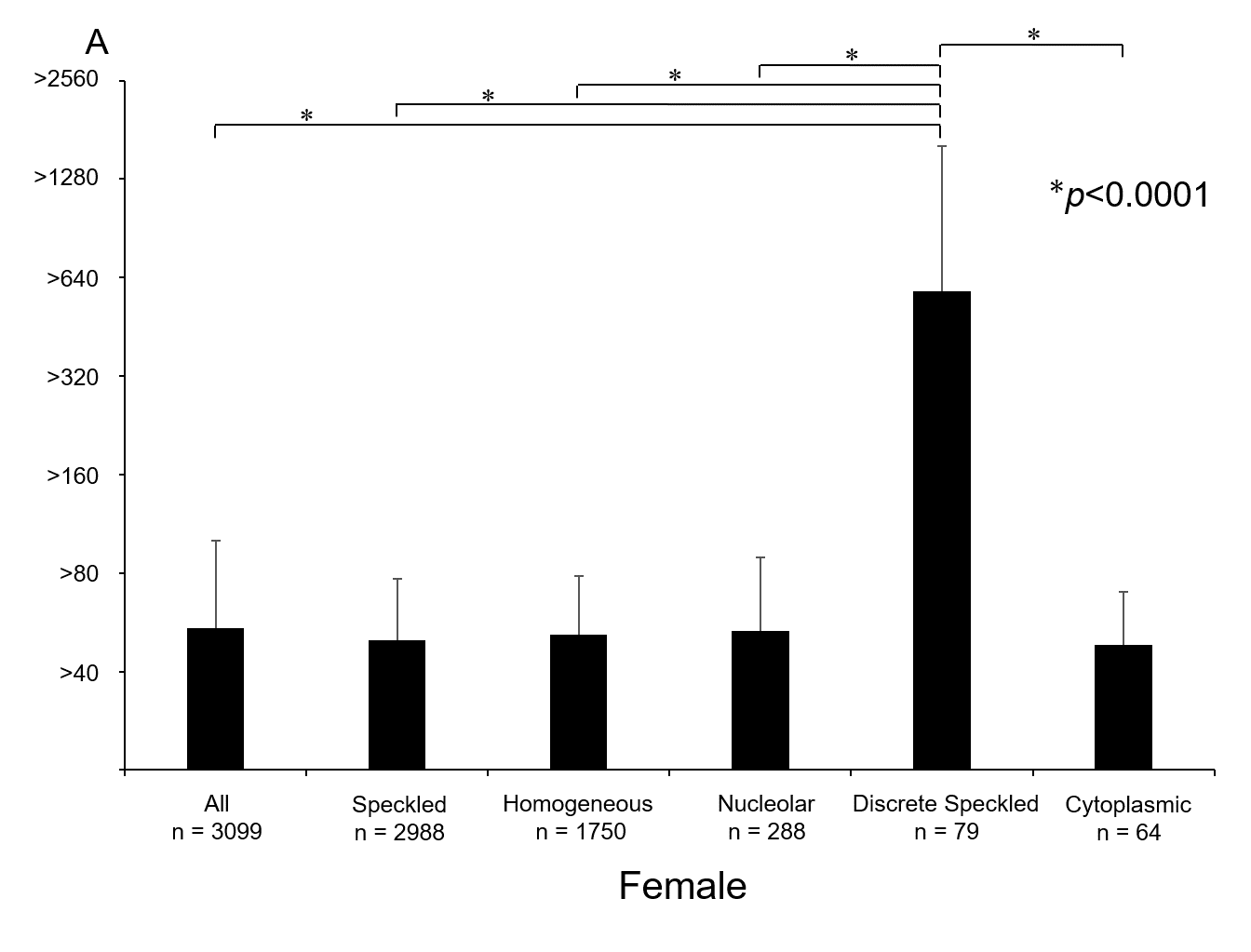
**

**
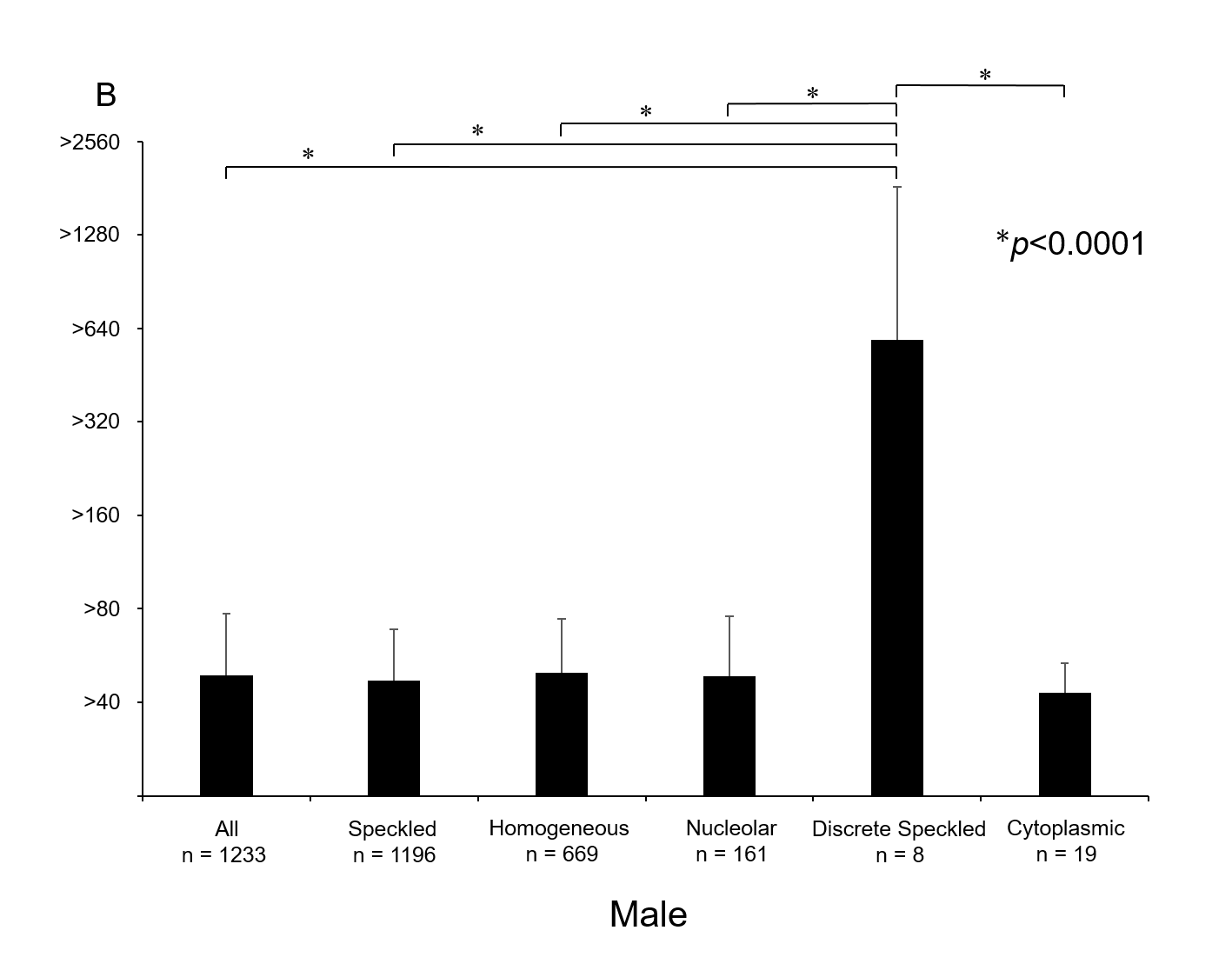
**

**Supplementary Table 1.** Chi-squared test between ACA and speckled pattern in healthy subjects and RA patients.

|  | Levels of ANA | | | | | | | P |
| --- | --- | --- | --- | --- | --- | --- | --- | --- |
|  | 40 | 80 | 160 | 320 | 640 | 1280 | 2560 |  |
| Healthy subjects |  |  |  |  |  |  |  | <0.0001 |
| ACA | 4 | 4 | 8 | 12 | 16 | 42 | 1 |  |
| Speckled | 3171 | 860 | 112 | 21 | 12 | 8 | 0 |  |
| RA |  |  |  |  |  |  |  | <0.0001 |
| ACA | 0 | 0 | 1 | 5 | 14 | 12 | 1 |  |
| Speckled | 349 | 170 | 94 | 54 | 25 | 11 | 2 |  |

ANA:anti-nuclear antibody, ACA: anti-centromere antibody, RA: rheumatoid arthritis

**Supplementary Table 2. Logistic regression analysis with response variate of ACA and independent variable of age in only female patients with RA.**

|  | Estimate | Standard Error | Chi-square | P Value |
| --- | --- | --- | --- | --- |
| Age | -0.062 | 0.020 | 9.86 | 0.0017 |

ACA, anticentromere antibody; RA, rheumatoid arthritis.

**Supplementary Table 3.** Logistic regression analysis showed significant associations between ACA and Raynaud’s phenomenon or secondary SS in condition with age and sex.

|  | Estimate | Standard Error | Chi-square | P Value |
| --- | --- | --- | --- | --- |
| Raynaud's phenomenon | 1.48 | 0.22 | 45.1 | <0.0001 |
| Secondary SS | 0.89 | 0.30 | 8.72 | 0.0032 |

SS, Sjögren’s syndrome.

**Supplementary Table 4.** Relationship between ACA-positivity and SE-positivity.

|  | KURAMA | | | |  | IORRA | | | |  | KURAMA + IORRA | | | |
| --- | --- | --- | --- | --- | --- | --- | --- | --- | --- | --- | --- | --- | --- | --- |
|  | ACA + (n=12) | ACA - (n=348) | p Value | OR (95% CI) |  | ACA + (n=11) | ACA - (n=470) | p Value | OR (95% CI) |  | ACA + (n=23) | ACA - (n=818) | p Value | OR (95% CI) |
| SE | 0.75 | 0.67 | 0.75 | 1.42 (0.41-6.55) |  | 0.82 | 0.70 | 0.39 | 1.94 (0.49-12.9) |  | 0.78 | 0.69 | 0.33 | 1.63 (0.64-4.98) |

KURAMA, Kyoto University Rheumatoid Arthritis Management Alliance; IORRA, Institute of Rheumatology, Rheumatoid arthritis; ACA, anti-centromere antibody; OR, odds ratio; 95% CI, 95% confidence interval, SE, shared epitope.

**Supplementary Table 5.** Association of HLA-DRB1 allelic groups with ACA-positivity.

|  | KURAMA + IORRA | | | |
| --- | --- | --- | --- | --- |
|  | ACA + (n=23) | ACA - (n=818) | P Value | OR (95% CI) |
| DR1 | 0.11 | 0.072 | 0.50 | 1.78 (0.58-4.56) |
| DR4 | 0.73 | 0.60 | 0.19 | 1.86 (0.73-4.76) |
| DR8 | 0.17 | 0.13 | 0.51 | 1.53 (0.51-4.59) |
| DR9 | 0.13 | 0.18 | 0.57 | 0.94 (0.34-2.29) |
| DR10 | 0.043 | 0.00061 | 0.034 | 9.63 (1.40-41.4) |
| DR11 | 0.00 | 0.021 | 0.32 | 0.00 (0.00-2.07) |
| DR12 | 0.087 | 0.10 | 0.85 | 0.87 (0.20-3.76) |
| DR13 | 0.043 | 0.067 | 0.67 | 0.64 (0.085-4.86) |
| DR14 | 0.00 | 0.11 | 0.090 | 0.00 (0.00-0.00) |
| DR15 | 0.043 | 0.27 | 0.014 | 0.12 (0.016-0.91) |
| DR16 | 0.043 | 0.010 | 0.035 | 4.49 (0.68-17.1) |

KURAMA, Kyoto University Rheumatoid Arthritis Management Alliance; IORRA, Institute of Rheumatology, Rheumatoid arthritis; ACA, anti-centromere antibody; OR, odds ratio; 95% CI, 95% confidence interval.

**Supplementary Table 6.** Statistical power of the current HLA data to detect a signal with p-value less than 0.01.

| Af | OR | | | | |
| --- | --- | --- | --- | --- | --- |
|  | 1.5 | 2.0 | 2.5 | 3.0 | 5.0 |
| 0.03 | 0.019 | 0.039 | 0.069 | 0.106 | 0.303 |
| 0.05 | 0.025 | 0.062 | 0.118 | 0.19 | 0.52 |
| 0.1 | 0.04 | 0.125 | 0.254 | 0.402 | 0.846 |
| 0.2 | 0.07 | 0.249 | 0.487 | 0.696 | 0.985 |
| 0.3 | 0.093 | 0.342 | 0.628 | 0.828 | 0.998 |
| 0.4 | 0.108 | 0.396 | 0.698 | 0.88 | 0.999 |
| 0.5 | 0.113 | 0.413 | 0.716 | 0.892 | 0.999 |

Af: allele frequency, OR: odds ratio
